# Inflammation Biomarkers of Advanced Disease in Nongingival Tissues of Chronic Periodontitis Patients

**DOI:** 10.1155/2015/983782

**Published:** 2015-05-07

**Authors:** Thiago Alvares da Costa, Marcelo José Barbosa Silva, Polyanna Miranda Alves, Javier Emílio Lazo Chica, Emilio Zorzo Barcelos, Max Antonio Alves Giani, Gustavo Pompermaier Garlet, João Santana da Silva, Virmondes Rodrigues Júnior, Denise Bertulucci Rocha Rodrigues, Cristina Ribeiro de Barros Cardoso

**Affiliations:** ^1^Instituto de Ciências Biológicas e Naturais, Universidade Federal do Triângulo Mineiro, Uberaba, MG, Brazil; ^2^Instituto de Ciências Biomédicas, Universidade Federal de Uberlândia, Uberlândia, MG, Brazil; ^3^Instituto de Ciências Tecnológicas e Exatas, Universidade Federal do Triângulo Mineiro, Uberaba, MG, Brazil; ^4^Departamento de Odontologia Clínica, Universidade de Uberaba, Uberaba, MG, Brazil; ^5^Departamento de Biologia Oral, Faculdade de Odontologia de Bauru, Universidade de São Paulo, Bauru, SP, Brazil; ^6^Departamento de Bioquímica e Imunologia, Faculdade de Medicina de Ribeirão Preto, Universidade de São Paulo, Ribeirão Preto, SP, Brazil; ^7^CEFORES, Universidade Federal do Triângulo Mineiro, Uberaba, MG, Brazil; ^8^School of Pharmaceutical Sciences of Ribeirão Preto, Department of Clinical Analyzes, Toxicology and Food Sciences, USP, Avenida do Café, s/n, 14040-903 Ribeirão Preto, SP, Brazil

## Abstract

Chronic periodontitis is a multifactorial inflammatory disease that affects supporting structures of the teeth. Although the gingival response is largely described, little is known about the immune changes in the alveolar bone and neighboring tissues that could indicate periodontal disease (PD) activity. Then, in this study we identified the ongoing inflammatory changes and novel biomarkers for periodontitis in the tissues directly affected by the destructive disease in PD patients. Samples were collected by osteotomy in 17 control subjects during extraction of third molars and 18 patients with advanced PD, in which alveoloplasty was necessary after extraction of teeth with previous extensive periodontal damage. Patients presented mononuclear cells infiltration in the connective tissue next to the bone and higher fibrosis area, along with increased accumulation of IL-17^+^ and TRAP^+^ cells. The levels of TNF-*α* and MMP-2 mRNA were also elevated compared to controls and a positive and significant correlation was observed between TNF-*α* and MMP-2 mRNA expression, considering all samples evaluated. In conclusion, nongingival tissues neighboring large periodontal pockets present inflammatory markers that could predict ongoing bone resorption and disease spreading. Therefore, we suggested that the detailed evaluation of these regions could be of great importance to the assessment of disease progression.

## 1. Introduction

Most conclusive evidences indicate that periodontal disease (PD) is not a conventional bacterial infection but is an inflammatory disease initiated by immune response against a group of microorganisms in susceptible hosts [1]. The consequent uncontrolled inflammation causes destruction of attachment structures, being the most significant reason of tooth loss in adults from different populations [2].

In affected tissues, the progression of local inflammation contributes to the clinical outcome of the disease [3]. Cytokines and chemokines lead to the migration of leukocytes to the periodontal tissues where these cells play an important role in pathogen destruction by releasing mediators in a local inflammatory response [4, 5]. Therefore, these and other mediators have been detected at elevated levels in the gingival crevicular fluid, saliva, and blood, thus being considered acceptable biomarkers for some aspects of PD [3, 6, 7]. Particularly,  proinflammatory cytokines such as Interleukin-1 beta (IL-1*β*) and tumor necrosis factor-alpha (TNF-*α*) have been associated with PD progression [8] and decline after periodontal treatment [9]. These cytokines are released by macrophages after bacterial infection or tissue injury and, in high concentration, stimulate the production and release of other inflammatory mediators [10]. Similarly, IL-17 is a proinflammatory cytokine produced by activated Th17 cells [11] that induce a distinct profile of effector cytokines which may exacerbate inflammation and tissue damage [12]. In fact, Th17 cells were found in human PD as well as IL-17 expression in the alveolar bone of these subjects, suggesting a causal link between inflammation and bone destruction [13]. Thus, while proinflammatory cytokines are potent inducers of bone resorption and inhibitors of bone formation [14], immunoregulatory mediators inhibit such proinflammatory signals and suppress the activation of metalloproteinases (MMPs) that mediate tissue destruction in PD [15].

MMPs are zinc-dependent endopeptidases capable of shredding extracellular matrix molecules. These enzymes are classified according to their catalytic domains or substrate specificity [16] and several studies have associated MMPs regulation with PD progression, once the control of synthesis and degradation of extracellular matrix defines levels of periodontal attachment [17]. Moreover, since MMPs production is potentiated in inflammatory conditions and these enzymes are related to PD susceptibility, the determination of the levels of inflammatory mediators in gingiva or biologic fluids became good indicators of the patient's periodontal disease status and activity [18, 19]. However, although how the inflammation develops in the gingival tissue of PD patients is largely described, little is known about the progression of this response to the alveolar bone underneath gingiva and how PD affects cytokine and MMPs expression in this tissue around the extensive periodontal pocket. Accordingly, the present study focused on the detection of the ongoing inflammatory changes in the bone and neighboring tissues directly affected by the destructive disease in PD patients.

## 2. Materials and Methods

### 2.1. Subjects

Seventeen (17) subjects with no periodontal or systemic diseases (controls) and 18 patients with advanced periodontal disease (PD) from the Periodontal Clinic of Uberaba University at Uberaba, Minas Gerais, Brazil, were selected for periodontal evaluation and sample collection. The subjects were submitted to anamnesis, clinical and periodontal exams, as proposed by the Periodontal American Association. All the clinical profile such as probing deep and clinical attachment was recorded for subjects classification into PD patients or controls (Table 1). Informed consents approved by the Local Ethics Committee on Human Research (protocol number 1230) were obtained from all the volunteers and the study was performed according to the Declaration of Helsinki, 1964.

### 2.2. Samples Collection

Bone biopsies were extracted from patients with advanced PD in which alveoloplasty was necessary before gingival suture, in cases of extraction of teeth with previous extensive periodontal damage. Therefore, the samples were obtained after maxillary or mandibular alveolar ridge regularization, with removal of minimal bone spicule from areas adjacent to the large bone resorption regions of the previously extracted teeth. Biopsies from control group were obtained during extraction of third molars that required osteotomy (*n* = 17). All samples used for gene expression analysis were first collected in RPMI medium supplemented with 10% of bovine fetal serum for transportation followed by washing in cold saline solution and storage into liquid nitrogen. Samples destined for histopathology study were washed in cold saline solution and fixed in PBS-formaldehyde 10%.

### 2.3. TRAP Analysis

Tartrate-resistant acid phosphatase (TRAP) activity was detected using a leukocyte-specific acid phosphatase kit (Sigma Aldrich, St. Louis, MO, USA) following the manufacturer's protocol. Briefly, samples were decalcified and embedded in paraffin and serial sections of 5 *μ*m were placed in glass slides. The sections were stained with TRAP solution for 1 hour at 37°C and counterstained with Mayer's Hematoxylin. Cells stained dark red to purple with more than three nuclei on the bone surfaces were considered TRAP^+^ and were counted per mm^2^ of tissue.

### 2.4. Real-Time Polymerase Chain Reaction Analysis

Quantitative polymerase chain reaction analysis (RT-PCR) of TNF-*α*, IFN-*γ*, IL-10, IL-4, and the metalloproteinases MMPs 1, 2, 3, and 9 mRNA expression was performed in the bone tissue samples. Primer sequences were designed using the software OligoPerfect Designer (Invitrogen, Carlsbad, CA, USA). All primers sequences were listed in Table 2. RNA was extracted according to the manufacturer's guidelines of RNA SV total RNA Isolation System kit (Promega, Madison, WI, USA) and the cDNA confection was performed with OligodT (Promega), dNTPs, reverse transcriptase enzyme M-MLV RT, and M-MLV buffer (Promega) in a PCR thermocycler PTC-100 (MJ Research, Waltham, MA, USA). The sequences of cDNA products were amplified in real-time PCR machine GeneAmp 7000 (Applied Biosystems, Foster City, CA, USA) using the reagent FastStart Universal SYBR Green Master (ROX) (Roche, Madison, WI, USA) and specific forward/reverse primers at 0.1 *μ*g/*μ*L. The default PCR condition was 2 minutes at 50°C, 10 minutes at 95°C and fifty cycles of 15 seconds at 95°C, 30 seconds at 58°C, and 30 seconds at 72°C. Sequence Detection Software version 1.3 (Applied Biosystems) was used to analyze data after amplification. Results were obtained as cycle threshold (Ct) values, which were normalized using the expression of the constitutive gene *β*-actin, following the equation 2^−ΔΔCt^, according to the Applied User's Bulletin #2, P/N 4303859 (Applied Biosystems).

### 2.5. Histopathology and Fibrosis Quantification

Biopsies were fixed with PBS-formaldehyde of 10% for 48 h and decalcified in PBS-EDTA of 0.5 M, during 30 days. Afterward, samples were dehydrated and embedded in paraffin. Sections (5 *μ*m) were stained with Hematoxylin and Eosin (HE) and analyzed by light microscopy. All images were captured with the digital video camera Evolution MP 5 (Media Cybernetics, Rockville, MD, USA) mounted on a light microscopy Eclipse 50i (Nikon, Melville, NY, USA) using the software Image-Pro Plus (Media Cybernetics). Morphometric analysis of fibrosis was performed using the ImageJ software (National Institutes of Health, Bethesda, MD). Fibrous connective tissues were stained with Picrosirius Red and the images were captured at ×400 (24 fields/sample) that measured 3705.82 *μ*m^2^. A grid with 100 points, each one representing 37.06 *μ*m^2^, divided each field. For each sample, 2400 points were analyzed totalizing 90192 *μ*m^2^. To calculate the percentage of collagen area, points coincident with stained area were counted. Fibrosis area was expressed as percentage of coincident points by total points counted. Points were checked by the formula of Hally, as described before [20].

### 2.6. Immunohistochemical Evaluation

For immunohistochemistry, deparaffinized sections were treated with 3% hydrogen peroxide in methanol for 10 min and incubated for 30 min at 90°C for antigen recovery. The blocking of the sections was performed using 2% bovine serum albumin incubated for 30 min at room temperature. Afterwards, they were individually incubated with anti-cytokine monoclonal antibodies specific for TGF-*β* (1 : 100) (R&D, Hopewell, NJ, USA) and IL-17 (1 : 20) (R&D). All antibodies were diluted in 2% BSA prior to use and incubated with the samples for 2 hours at 37°C. A secondary step was performed using biotinylated anti-mouse Ig, anti-rabbit Ig, and anti-goat Ig from Link System 002488 (Dako, Carpinteria, CA, USA) for 30 min at 37°C. After being washed, the sections were incubated with streptavidin-peroxidase conjugated (Dako) for 30 min. The reactions were detected with diaminobenzidine (DAB) (Sigma Aldrich). The sections were counterstained with Mayer's Hematoxylin. For histopathological analysis, the number of positive cells for each cytokine was counted in 20 fields at a magnification of ×400, in a predetermined area of 0.091575 mm^2^. The density of positive cells was expressed as the number of cells per mm^2^.

### 2.7. Statistical Analysis

Graph Pad InStat and Prism statistical programs were used for analysis (GraphPad, San Diego, CA, USA). In case of normal distribution, the Independent *t*-test was used while the nonparametric Mann-Whitney test was used in non-Gaussian sample distribution. Correlation was verified using Spearman test. Data were considered statistically significant when *p* < 0.05.

## 3. Results

### 3.1. Clinical Findings

First, we performed a clinical periodontal examination and an analysis of the status of the volunteers enrolled in the study to better classify the individuals into PD or control group and to verify if these findings could be related to the next evaluations. Data showed that both groups were balanced for gender and were different for age distribution, since chronic periodontitis was more prevalent in older individuals. Regarding tobacco use, 4 patients from PD group were smokers, a habit known to be directly related to PD progression. The probing depth was not different between control and PD group (*p* > 0.05) since there was extensive clinical attachment loss with gingival recession in PD patients, thus accounting to their classification into the advanced PD group (Table 1).

### 3.2. Histological Features Indicate Local Inflammation in Periodontium Area

**Figure 1:**
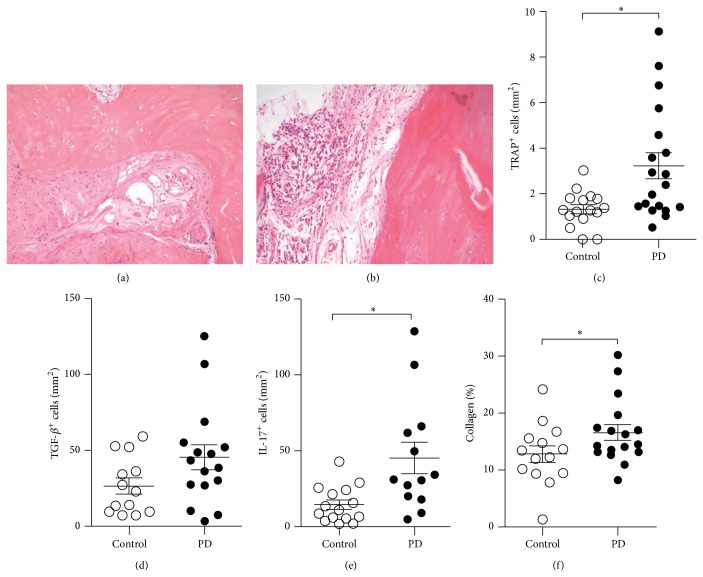
Histopathological analysis and cytokine and collagen production in tissues extracted from control and periodontal disease (PD) patients. Samples were fixed, decalcified, embedded in paraffin, and stained with Hematoxylin and Eosin (HE) or tartrate-resistant acid phosphatase (TRAP) staining protocol. (a) and (b) represent the HE slides from bone and adjacent tissue of control and PD group at 200x, respectively. (c) depicts the number of TRAP positive cells in the bone and adjacent tissue of control (*n* = 16 and PD (*n* = 19) subjects. Immunohistochemical staining is shown for TGF-*β* (d) and IL-17 (e) for control (*n* = 13) and PD (*n* = 15) subjects. In (f), the percentage of Picrosirius stained areas for collagen quantitation in control and PD group, as described in Section 2.^∗^ *p* < 0.05.

Next, to verify if the periodontal and especially the bone tissue adjacent to the areas of extensive bone resorption in advanced PD presented any sign of inflammation, we performed histopathological evaluation of the HE stained biopsies. Results showed that control subjects presented a normal aspect of bone tissue with osteocytes and osteoblasts at the edge of bone matrix with no signs of alteration (Figure 1(a)). The connective tissue next to it did not present any significant inflammatory alteration. In contrast, samples from PD group presented a focally distributed and mild inflammatory process composed predominantly by mononuclear cells with the presence of lymphocytes and plasma cells, besides a few neutrophils. The bone tissue did not present any significant alteration (Figure 1(b)). Then, since osteoclast-mediated bone resorption may be driven by local inflammation, we next aimed to verify if it could be an indicative of ongoing bone damage. In fact, we found increased numbers of TRAP-positive cells in samples from PD group when compared to controls (Figure 1(c)), indicating that the inflammatory process was able to trigger osteoclast differentiation in periodontitis patients.

### 3.3. Cytokines and Collagen Balance in PD Progression

Previous studies from our group showed that advanced PD is characterized by elevated levels of TGF-*β* and IL-17 mRNA, which may be related to disease progression [13, 21]. Then, in the present study, immunohistochemical analysis revealed that both groups produced TGF-*β* and IL-17 cytokines (at protein level) in the periodontium biopsies. PD subjects presented a slight augment in the density of TGF-*β* positive cells (Figure 1(d)) and a significant increase was observed in the number of IL-17 positive cells in comparison to the control group (Figure 1(e)). Furthermore, since the collagen matrix in the bone and connective tissue of the periodontium are one of main targets in periodontitis destruction, we determined the collagen percentage in the biopsies stained by Picrosirius Red. Surprisingly, although PD subjects presented microscopic inflammation and osteoclast accumulation suggestive of tissue damage, a higher collagen deposition was found in this group, as observed in Figure 1(f).

### 3.4. Cytokines and Metalloproteinase mRNA Expression

**Figure 2:**
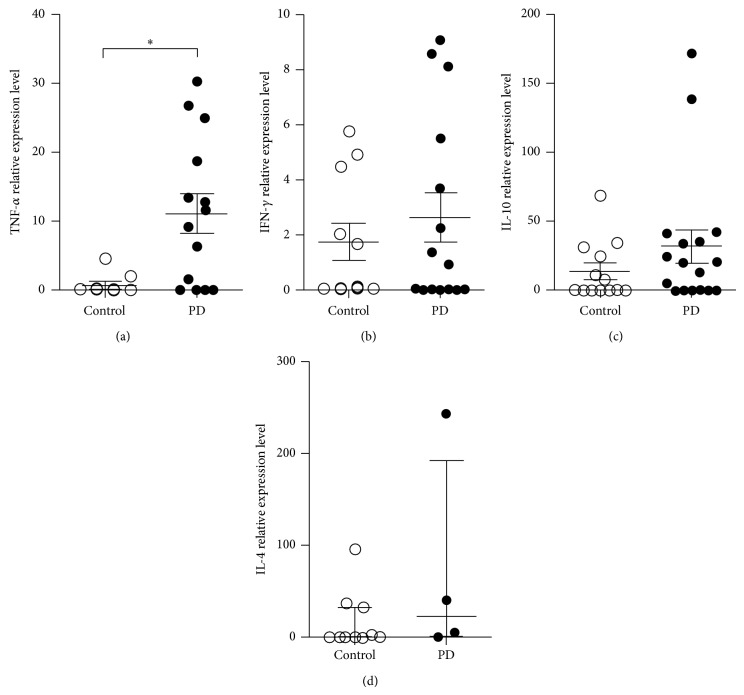
Cytokine expression in alveolar bone tissue of control and periodontal disease (PD) patients. Total RNA extracted from bone biopsies of PD and control group was subjected to qRT-PCR, SYBR green method. RNA expression of TNF-*α* (a), IFN-*γ* (b), IL-10 (c), and IL-4 (d) is presented as the intensity of expression of the individual mRNAs, with normalization to *β*-actin, using the cycle threshold method. Data are the mean ± SEM results for patients with PD and control subjects tested individually. The results shown are from one experiment representative of three. ^∗^ *p* < 0.05.

**Figure 3:**
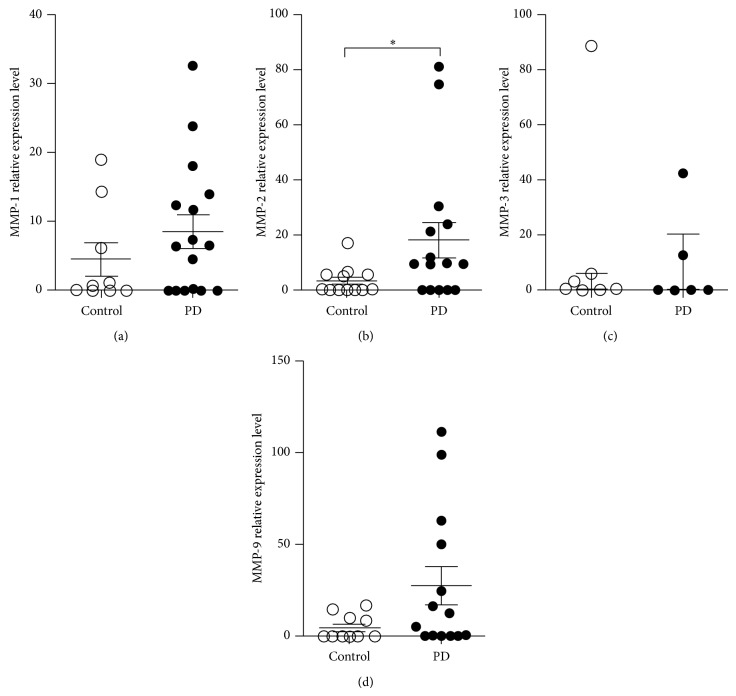
Metalloproteinases (MMPs) expression in alveolar bone tissue of control subjects and periodontal disease (PD) patients. Total RNA extracted from bone biopsies of PD and control groups was subjected to qRT-PCR, SYBR green method. RNA expression of MMP-1 (a), MMP-2 (b), MMP-3 (c), and MMP-9 (d) is presented as the intensity of expression of the individual mRNAs, with normalization to *β*-actin, using the cycle threshold method. Data are the mean ± SEM results for patients with PD and control subjects tested individually. The results shown are from one experiment representative of three. ^∗^ *p* < 0.05.

**Figure 4:**
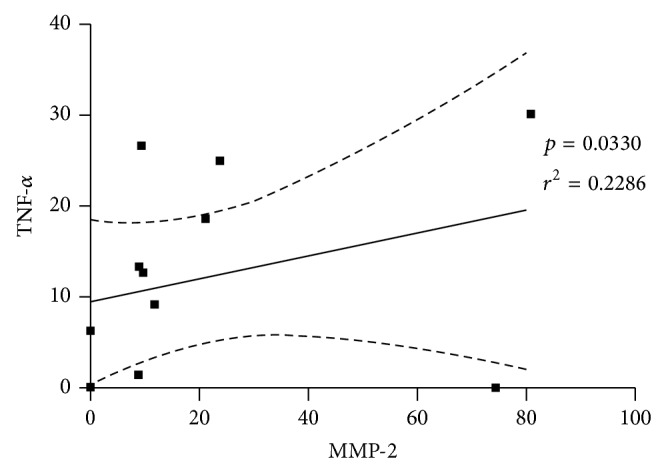
Correlation analysis between TNF-*α* and MMP-2 mRNA from bone biopsies of control and periodontal disease (PD) patients. The samples were subjected to qRT-PCR, SYBR green method. Data are reported for control (*n* = 8) and PD group (*n* = 12) tested individually. *p* = 0.0036, *r*_*s*_ = 0.6190, Spearman test.

Once bone resorption is a result of the balance between anti- and proinflammatory mediators that may activate tissue destructive enzymes, we assessed the expression of cytokines and metalloproteinases (MMPs) in the bone tissue of PD and control samples. The real-time PCR assays revealed that the levels of IFN-*γ*, IL-10, and IL-4 mRNA were not altered in the samples of subjects relative to controls (Figures 2(b)–2(d)). However, there was a strikingly elevated expression of TNF-*α* mRNA in subjects with PD compared to control group (Figure 2(a)). Importantly, this increase in the inflammatory cytokine TNF-*α* was accompanied by augmented expression of MMP-2 mRNA (Figure 3(b)) and there was a tendency towards elevated expression of MMP-1 and MMP-9 in PD patients (Figures 3(a) and 3(d)). MMP-3 was not different between the studied groups (Figure 3(c), *p* > 0.05). Furthermore, a positive and significant correlation was observed between TNF-*α* and MMP-2 mRNA expression when all the subjects of the study were evaluated together (Figure 4, *p* = 0.0036; *r*_*s*_ = 0.6190). Altogether, these data suggested that the local immune alterations that culminate in tooth loss are not restricted to gingiva. The periodontal or bone tissue next to the extensive areas of resorption presents relevant inflammatory findings indicative of periodontitis destruction and progression in susceptible individuals.

## 4. Discussion

The data presented in this study obtained from examination of sites close to areas with extensive periodontal damage showed that, as expected, inflammation in advanced PD is not restricted to gingiva. Otherwise, tissues like alveolar bone that is clinically undergoing a resorption process present relevant findings that could be considered inflammatory biomarkers for disease development in susceptible patients. Although predictable, this is the first study that showed the immune response related to periodontitis evolution directly in the damaged bone and neighboring area.

Interestingly, our patients with advanced PD presented a number of features characteristics of local inflammation and some of them were smokers, a habit known to be classically involved in PD progression. In spite of this, it did not seem to interfere in the disease spreading to bone tissue, since the inflammation markers analyzed were not differentially expressed by these individuals.

As a next approach, we examined whether PD could result in histological changes in the bone resorbing areas close to the large periodontal pocket. The histological parameters indicated a mild inflammatory process composed predominantly by mononuclear cells. Also, this mild inflammation associated with TRAP^+^ cells could be an indicative of microbial presence and an active process of tissue damage, though not at the same extent yet as that observed in the large periodontal pocket, that led to the tooth loss. Corroborating our data, previous studies showed that pathogenic microorganisms might colonize different sites and then spread local inflammation in case of uncontrolled pathogen burden [22, 23].

In fact, the key feature of PD is alveolar bone loss. The integrity of bone tissue depends on the balance between bone formation by osteoblasts and bone resorption by osteoclasts [24, 25]. In this scenario, several proinflammatory cytokines were identified as key molecules contributing to the destruction of periodontal tissue, including IL-1, TNF-*α*, IFN-*γ*, and IL-6 besides RANKL [26, 27]. Thus, in view of our results, it is possible that the higher TNF-*α* mRNA found in our patients may have acted as an important osteoclastogenic factor by inducing the local osteoclast differentiation that could culminate in bone resorption.

We also revealed that the number of IL-17^+^ cells was higher in PD regions than in control healthy tissues. Such cells could be CD4^+^, CD8^+^, *γδ* ^+^, neutrophils, natural killer cells, or others [28–32]. A previous study demonstrated that patients with chronic periodontitis have Th17 cells as well as IL-17 expression in inflamed periodontal tissue, indicating the possible involvement of this cytokine in periodontal disease [13]. Indeed, Th17 cells could exacerbate inflammatory periodontal disease by inducing metalloproteases or inflammatory mediators by gingival fibroblasts [33]. In this context, our results suggested the involvement of Th17 cells in the inflammatory events triggered in the periodontal disease affected areas. Besides, IL-17 could be capable of inducing RANKL, the main stimulatory factor for the differentiation and activation of osteoclasts in bone inflammatory diseases like rheumatoid arthritis and PD [34, 35]. Then, the higher number of osteoclasts in the areas evaluated could also be related to the presence of IL-17^+^ cells.

The inflammatory process in PD leads to the degradation of extracellular matrix, mainly collagen, by MMPs overproduction that may be induced by cytokines released from resident and inflammatory cells [36, 37]. However, during the healing process that follows inflammation, collagen is also newly produced for extracellular matrix remodeling [38]. It is possible to observe such phenomena in periodontal tissue after periodontal disease treatment [37]. Therefore, the increased collagen deposition observed in our patients could result from a repair response to the initial inflammation triggered nearby the extensive bone resorption areas. In fact, the number of TGF-*β* ^+^ cells was slightly higher in PD group compared to controls. Nevertheless, this cytokine could play a dual role in PD, by controlling inflammation or inducing the differentiation of Th17 response [13, 21].

Most strikingly, as discussed before, levels of TNF-*α* were significantly augmented in bone tissue from PD group. As TNF-*α* is important for osteoclastogenesis, it is possible that this factor initiates osteoclast differentiation in subclinical inflammatory process. Also, TNF-*α* is produced in response to periodontopathogens, inducing the microbicidal activity of phagocytic cells, and, in the absence of the subunit p55 of TNF-*α* receptor, mice infected with* Aggregatibacter actinomycetemcomitans* presented less bone resorption and inflammatory response, besides increased number of bacteria [39]. Therefore, the higher levels of TNF-*α* in our PD group could indicate the presence of pathogens in periodontal environment, besides accounting for the higher number of osteoclasts in these patients [40, 41].

During active periodontitis, degradation of gingival connective tissues, mainly collagen, is due to the expression of MMPs* in situ* by inflammatory and resident cells such as fibroblasts and epithelial or endothelial cells [33, 37]. However, the evaluation of MMPs directly in the bone resorbing areas adjacent to the large periodontal pocket, as well as their role in the disease outcome, is unclear. Our subjects with PD presented a mild inflammatory infiltration of cells and the expression of MMP-2 mRNA was almost five times higher in samples from this group. This local infiltrate could be able to produce MMP-2, based on previous results indicating that TNF-*α* stimulates the secretion of active MMP-2 [42, 43], thus resulting in degradation of collagen types I, II, and III. MMP-2 expression in biopsies of subjects with PD could suggest a possible biological alteration in the turnover of connective tissue and a consequent active ongoing process of bone resorption. Also, the degradation of collagen by MMP-2 could produce peptides important for recruitment of mononuclear and polymorphonuclear cells that could later amplify the inflammatory process.

In our study, the mRNA for TNF-*α* was also correlated with MMP-2. Although we did not evaluate MMP-2 activity* in situ*, we might hypothesize that the production of TNF-*α* could upregulate membrane type metalloproteinase-1 (MT1-MMP), which is able to activate the latent form of MMP-2 [44], thus breaking collagen in PD sites. Such phenomenon could be confirmed by higher percentage of collagen areas, indicating a remodeling process that follows collagen degradation. Indeed, repeated exposure to low or moderate LPS concentration induced augmented cardiac fibrosis along with elevated MMPs expression (including MMP-2) in mice heart, thus indicating that a subclinical inflammatory response may be able to trigger tissue collagen response and alter MMPs balance [45]. However, future studies are still necessary to better elucidate this hypothesis.

## 5. Conclusions

Tissues neighboring large periodontal pockets present inflammatory markers such as IL-17, TNF-*α*, MMP-2, and osteoclasts accumulation, which could be predictors of local bone resorption and disease spreading. These results support the importance of a more detailed evaluation of these regions other than gingiva, since the local immune response may represent sentinel factors relevant for disease outcome.

## Figures and Tables

**Table 1:** Clinical profile of subjects from periodontal disease (PD) and controls.

	Control (*n* = 17)	PD (*n* = 18)
Gender (M/F)	9/8	9/9
Frequency	52.94%/47.06%	50%/50%
Age group (years, mean ± SD)	29.06 ± 9.69	46.28 ± 13.41
Smokers	—	4
Mean probing depth (mm)	2.22	3.55
Clinical attachment loss (mm, mean ± SD)	—	5.18 ± 1.40

M: male; F: female; SD: standard deviation.

**Table 2:** Human primers sequences for real-time PCR used in this study.

Genes	Sequence (5′→3′)
*β*-actin	S - TGA CTC AGG ATT TAA AAA CTG GAA
	AS - GCC ACA TTG TGA ACT TTG GG

TNF-*α*	S - TTC TGG CTC AAA AAG AGA ATT G
	AS - TGG TGG TCT TGT TGC TTA AAG

IFN-*γ*	S - GAG AAC CCA AAA CGA TGC A
	AS - ACT TCT TTG GCT TAA TTC TCT CG

IL-4	S - GAA GGA AGC CAA CCA GAG TA
	AS - GAT CGT CTT TAG CCT TTC CA

IL-10	S - TTC CCT GAC CTC CCT CTA ATT
	AS - GCT CCC TGG TTT CTC TTC CTA A

MMP-1	S - GAT TGA AAA TTA CAC GCC AGA T
	AS - TCT CAA TGG CAT GGT CCA

MMP-2	S - ACT GTT GGT GGG AAC TCA GA
	AS - TTG TTG CCC AGG AAA GTG

MMP-3	S - AAG GAT ACA ACA GGG ACC AA
	AS - CAG TGT TGG CTG AGT GAA AG

MMP-9	S - TCA CTT TCC TGG GTA AGG AGT A
	AS - TGT CAA AGT TCG AGG TGG TAG

S: sense; AS: antisense.
